# “None of it was especially easy”: improving COVID-19 vaccine equity for people with disabilities

**DOI:** 10.17269/s41997-022-00621-z

**Published:** 2022-04-13

**Authors:** Jennifer C. H. Sebring, Gabriela Capurro, Christine Kelly, Cynthia G. Jardine, Jordan Tustin, S. Michelle Driedger

**Affiliations:** 1 Department of Community Health Sciences, University of Manitoba, S113-750 Bannatyne Ave, Winnipeg, MB R3E 0W3 Canada; 2 Faculty of Health Sciences, University of Fraser Valley, 45190 Caen Ave, Chilliwack, BC V2R 0N3 Canada; 3 School of Occupational and Public Health, Ryerson University, 350 Victoria Street, Toronto, ON M5B 2K3 Canada

**Keywords:** Immunization, Disabilities, Inequity, Pandemic, Immunisation, invalidités, iniquité, pandémie

## Abstract

**Objectives:**

Our study aimed to (1) identify barriers to equitable access to COVID-19 vaccines for Canadians with disabilities and (2) present recommendations made by study participants to improve immunization programs in terms of inclusivity and equitable access.

**Methods:**

We invited Manitobans living with disabilities to participate in online focus groups. Focus groups were conducted across multiple disability experiences, although one focus group was advertised explicitly as offering simultaneous American Sign Language interpretation to encourage people who are d/Deaf or hard of hearing to participate. Participants were asked about their perspectives on the management of COVID-19 public health measures and vaccination program rollout. Participants were also asked about barriers and facilitators of their vaccination experiences and if they had recommendations for improvement.

**Results:**

The participants identified three areas where they encountered routine barriers in accessing the COVID-19 vaccines: (1) vaccine information and appointment booking, (2) physical access to vaccination clinics, and (3) vaccination experience. While participants identified specific recommendations to improve vaccine accessibility for people with disabilities, the single most crucial advice consistently identified was to involve people with disabilities in developing accessible immunization programs.

**Conclusion:**

Meaningful engagement with people living with disabilities in immunization program planning would help ensure that people with disabilities, who already face significant challenges due to COVID-19, are offered the same protections as the rest of the population. These recommendations could be easily transferred to the administration of other large-scale immunization campaigns (e.g., influenza vaccines).

## Résumé

**Objectifs:**

Notre étude visait à (1) identifier les obstacles à un accès équitable aux vaccins contre la COVID-19 pour les Canadiens vivant avec un handicap; et (2) présenter les recommandations faites par les participants à l’étude pour améliorer les programmes de vaccination en termes d’inclusivité et d’accès équitable.

**Méthodes:**

Nous avons invité les Manitobains vivant avec un handicap à participer à des groupes de discussion en ligne. Les groupes de discussion ont été organisés pour divers types de handicap, bien qu’un groupe de discussion ait été explicitement annoncé comme offrant une interprétation simultanée en langue des signes américaine pour encourager les personnes sourdes ou malentendantes à participer. Les participants ont été interrogés sur leurs points de vue sur la gestion des mesures de santé publique contre la COVID-19 et le déploiement du programme d’immunisation. Les participants ont également été interrogés sur les obstacles et les facilitateurs de leurs expériences de vaccination et s’ils avaient des recommandations d’amélioration.

**Résultats:**

Les participants ont identifié trois domaines dans lesquels ils ont rencontré des obstacles récurrents pour accéder aux vaccins contre la COVID-19 : 1) l’information sur les vaccins et la prise de rendez-vous, 2) l’accès physique aux cliniques de vaccination, et 3) l’expérience de la vaccination. Bien que les participants aient formulé des recommandations spécifiques pour améliorer l’accessibilité des vaccins pour les personnes handicapées, le conseil le plus crucial systématiquement identifié a été d’impliquer les personnes vivant avec un handicap dans l’élaboration de programmes d’immunisation accessibles.

**Conclusion:**

Un engagement significatif envers les personnes handicapées dans la planification du programme d’immunisation aiderait à garantir que les personnes vivant avec un handicap, qui sont déjà confrontées à des défis importants en raison de la COVID-19, bénéficient des mêmes protections que le reste de la population. Ces recommandations pourraient être facilement transférées à l’administration d’autres campagnes de vaccination à grande échelle (par exemple, le vaccin contre la grippe).

## Introduction

The COVID-19 pandemic transformed and continues to transform people’s lives in innumerable ways, causing significant disturbances in almost every aspect of life. Along with social and economic shutdowns, public health authorities exhort citizens to adopt personal protective behaviours (i.e., mask-wearing, social distancing, quarantining/isolation), seek testing if symptomatic or potentially exposed, and get vaccinated as soon as possible. However, health crises like the COVID-19 pandemic do not affect all people equally. Through the introduction of policies and programs alongside existing systemic inequities, some groups are *made* more vulnerable (Tremain, 2020) to adverse outcomes, including people living with disabilities (PLWD).

In this article, we define PLWD as those who have long-term physical, mental, intellectual, or sensory impairments which, in interaction with various barriers, may hinder their full and effective participation in society on an equal basis with others (United Nations, 2006). Such barriers can be physical, linguistic, geographic, income-related, and cultural. We intentionally adopt a broad definition in this article to include those who have chronic illnesses, chronic pain, and mental health conditions alongside those who identify as having an impairment, emphasizing the role of barriers in shaping individual and collective experiences of disability. The lack of social supports available to address barriers contributes to PLWD having poorer health outcomes than the general population (Horner-Johnson et al., 2013; Lebrasseur et al., 2021; Tracy & McDonald, 2015). Relatedly, PLWD are forced to grapple with the ways their well-being is embedded in social structures, infrastructures, and attitudinal environments that can be discriminatory and indifferent to their experiences (Powell, 2021).

The COVID-19 pandemic exacerbates many existing barriers for PLWD, who still require care support and access to health services that have been reduced or even suspended (Lebrasseur et al., 2021; Smith et al., 2021). Protective protocols (e.g., masking, social distancing) complicate access to transportation services, public health service infrastructure, and communication (e.g., lip reading, ASL interpretation). PLWD may also have particular comorbidities that place them at increased risk for more severe outcomes if infected with COVID-19, such as diabetes or respiratory or heart conditions (Armitage & Nellums, 2020; Lunsky et al., 2021).

COVID-19 vaccination programs have been widely adopted to contain the spread of the virus (Dhama et al., 2021); however, vaccine access is inequitable (Burki, 2021). In Canada, vaccine priority groups were established (National Advisory Committee on Immunization, 2020), and in Manitoba, elderly living in long-term care and health care workers were initially prioritized (Manitoba, 2021). In March 2021, vaccination eligibility was expanded to include Manitobans 50 to 64 years old, and 30 to 64 years old among First Nations populations, who have high-risk conditions that increase their chances of negative outcomes (Unger, 2021). In addition, the creation of two priority groups allowed individuals with specific health conditions or those using frequent home care services to receive the vaccine ahead of the general population (Unger, 2021). However, PLWD face many challenges when trying to access COVID-19 immunizations, such as booking mechanisms and transportation, and tasks that were challenging before the pandemic are further disrupted by COVID-19 protective protocols (Women in Global Health, 2020). Last, COVID-19 compounds everyday stress and anxiety while PLWD navigate perceived risk of infection during everyday interactions in public spaces, or when care-workers bring the “outside” into client homes (Lourens & Watermeyer, 2021).

As Canadian provinces prepared their COVID-19 vaccination programs, health experts and disability-focused community leaders recommended creating them based on principles of equity, human rights, social justice, and inclusivity (Armitage & Nellums, 2020; Ismail et al., 2020). Developing public health responses that are inclusive of PLWD need to be based on the tenets of disability justice (Berne et al., 2018), something that is only slowly gaining traction in public health spheres (Gaventa et al., 2021; Guidry-Grimes et al., 2020). Disability justice is a radical and transformative movement that takes up the limitations of earlier disability rights activism (Lamm, 2015; Lord, 2010; Mingus, 2011), centring the experiences of multi-marginalized people living with disabilities, namely people of colour and queer, transgender, Black, and/or Indigenous people.

While community involvement is increasingly practiced in public health decision-making (Haldane et al., 2019), disability justice demands a *leadership* role for affected communities. In practice, this means moving beyond consultation to shift decision-making power squarely with the community. Further, disability justice is committed to intersectional (Crenshaw, 1990) access and reflects cross-disability solidarity. Improving access means we must consider the multiplicities of disability experiences and how ableism is implicated and mutually constituted by other systems of oppression, including classism, racism, and colonialism. Disability justice widens our understanding of equity and access and addresses barriers experienced not only by PLWD but also by a multitude of communities who are vulnerable to the policies enacted during COVID-19.

The literature on how PLWD have experienced the COVID-19 pandemic thus far is scarce (cf. Lebrasseur et al., 2021; Stienstra et al., 2021). Critical assessments of the needs of PLWD must address challenges faced by PLWD before and during the pandemic and improve management of current and future Public Health Emergencies of International Concern (PHEIC) (World Health Organization, 2005). Our study examines the general experiences of PLWD in Manitoba during the COVID-19 pandemic and, more specifically, barriers to vaccine access. We offer several recommendations to improve vaccine equity for PLWD.

## Methods

We conducted six online focus groups with Manitobans with disabilities to get their perspectives on the COVID-19 public health measures and vaccination program rollout between May 27 and June 11, 2021. This project is part of a larger project on COVID-19 management strategies across Canada (Driedger et al., 2020).

Following principles of community-based participatory research (Israel et al., 1998), we first consulted with four local and national cross-disability organizations^1^ run by PLWD to assess the relevance of our research. Representatives from these organizations provided feedback on the focus group questions and agreed to circulate recruitment material. We used several recruitment strategies, including social media, Kijiji, and email distribution lists provided by the organizations. Interested participants received a unique link to a consent form and a short survey. We allowed caregivers of PLWD to participate, up to a maximum of 25% of the total participants. The survey provided descriptive characteristics of our sample population^2^, and gauged individual perceptions of COVID-19, vaccine acceptance, vaccine rollout, and overall pandemic response. As the consent form and survey were both online activities, alternative options (e.g., receiving documents in a format compatible with their screen reader, responding to items orally over the phone) were provided to accommodate all participant needs. We were interested in cross-disability perspectives and did not seek to hold focus groups specific to individuals experiencing the same type of disabilities. However, we did advertise a specific date to provide American Sign Language (ASL) interpretation.

A total of 30 participants completed the pre-focus group consent and survey. Of these participants, 23 could attend one of the six focus groups (see Table 1). The majority of participants identified as having a chronic illness or chronic pain. However, there was representation across all disability types, including physical disabilities, intellectual disabilities, mental health conditions, and sensory impairments. The online nature of the focus groups meant that participants were drawn from Winnipeg and other smaller urban and rural communities where internet access is more reliable.

**Table 1:** Socioeconomic and demographic characteristics of participants, *N* = 23

Characteristic	Count (%)
Gender	
Male Female	6 (26.1) 17 (73.9)
Age group (years)	
18–24 25–30 31–34 35–40 41–48 49–54 55–60 61–68	3 (13.0) 3 (13.0) 1 (4.3) 2 (8.7) 4 (17.4) 3 (13.0) 4 (17.4) 3 (13.0)
Marital status	
Single (never married) Married or common law Divorced, separated, or widowed	10 (43.5) 6 (26.1) 7 (24.1)
Number of children under 18 years in household	
0 1 2	18 (78.3) 4 (17.4) 1 (4.3)
Education	
High school Some college/university College/university degree	5 (17.2) 3 (13.0) 15 (65.2)
Income ($CAN)*	
Under $50,000 $50,000–$74,999 $75,000–$99,999 $100,000–$149,000	18 (78.3) 1 (4.3) 1 (4.3) 2 (8.7)
Type of disability	
Mental health condition Chronic illness/pain Sensory impairment (visual, hearing) Intellectual disability Physical impairment	5 (21.7) 11 (47.8) 4 (17.4) 2 (8.7) 1 (4.3)
Race (based on self-identification)	
White Person of colour Métis	17 (73.9) 3 (13.0) 3 (13.0)
Geographic location	
Winnipeg Outside of Winnipeg	19 (80.6) 4 (17.4)

*1 participant selected “Prefer not to answer.”

The project lead, a qualitative researcher with over 20 years of experience, moderated the focus group discussions and led the analysis. Two research assistants observed each focus group and took detailed notes to quickly generate a summary report for participants. The focus group discussion questions explored participant perceptions of COVID-19, including their assessment of public health guidelines and conflicting or confusing messaging. Participants were invited to share their perceptions, willingness, and experience in getting COVID-19 vaccines. Conversations ended by asking participants to imagine they had the Chief Provincial Public Health Officer or Premier sitting across from them and to provide them with constructive feedback. The discussions lasted two hours, and participants received an honorarium of $70 for their time.

All focus groups were audio-recorded, transcribed verbatim, and audio-verified. Transcripts were uploaded for analysis to the qualitative analysis software NVivo12. To identify participants in the transcripts and publications, we used the name they requested. We adapted our existing coding framework to capture the views of PLWD. The principal codes^3^ corresponded to the questions asked in the focus groups. Codes were then added for specific barriers or facilitators in accessing COVID-19 information sources and vaccination programs, and suggestions for how these could be improved for PLWD. Two team members coded the transcripts, and two coding tests were performed with a third member of the research team to ensure inter-coder reliability. Our kappa coefficient score was 0.90. Our analysis explored the data intersectionally to identify if participant narratives of their experiences varied by type(s) of disability and other relevant sociodemographic characteristics (Richards, 2009). This study received ethics clearance from the University of Manitoba Health Research Ethics Board (H2020:510 linked to H2020:164) and the Research Ethics Board of Ryerson University (2020:445).

## Results

### Barriers to accessing the COVID-19 vaccine

Participants identified a series of equity-related barriers regarding the COVID-19 vaccine: accessing information about COVID-19 vaccines and how to book appointments; difficulties accessing vaccination sites; and other obstacles that made the overall vaccination process challenging. Participants noted that most of these barriers could have been easily avoided with proper planning.

#### Vaccine information and appointment booking

Accessing information about the COVID-19 vaccine proved challenging for many participants, who had problems navigating the provincial and local websites due to visual impairments or inability to find relevant information. One participant with a visual impairment explained that she needed to ask a relative for help navigating a particular website and printing her consent form. She noted “their website isn’t the most accessible, even for looking for places that are offering vaccines, the vaccine finder (…) There’s lots of pop-ups and sidebars that for those who use screen readers, it’s not the most successful” (Ana). Other participants with hearing impairments recalled trying to keep informed by watching provincial leaders’ press conferences; however, the lack of ASL interpreters during the broadcasts made this difficult. Even when provided, the image of the ASL interpreter was too small for effective visual communication.

[I]f there’s an interpreter there, they might hire them for the [press] conference, but they’re not on the TV, so we can’t see them, they’re not visible. They might be over in a different area, or they’re quite small on the screen, so we can’t see them as well. […] Or it might be a hearing interpreter, and the deaf community is preferring to have a deaf interpreter on screen, because they’re more fluent than the hearing interpreter. It’s been quite a process to try to get those proper accessibility things in place (Terri).

Other participants found it was easier to access press conference information online through re-packaged news articles, which were accessible through screen readers. Some participants mentioned having difficulty booking a vaccine, particularly those with a visual impairment or who are d/Deaf or hard of hearing. For example, one person who is d/Deaf booked their vaccine online only to receive a message indicating they would get a confirmation phone call. The participant explained that this caused them great anxiety because they can never be certain if the call will be in an appropriate format. Another participant mentioned having problems booking vaccine appointments for her relatives, who are also PLWD, because the system would not allow third-party bookings:

So, my family, grandparents and parents included, did not get their vaccine until we were able to have a third party book and book online. So, it was a slow process to get to that point, because it was very rough at first (Jennifer).

Other participants explained they had extreme difficulty finding information specific to PLWD in general or for their specific disability. They noted that most of the information provided online is too broad. A participant explained that government information “is not really specific for me, so it’s not really helpful for my understanding of what to do. I know the information is there, but it’s not very helpful for me” (Terri). Similarly, a participant’s caregiver mentioned they could not find clear information on when the participant, who had a priority health condition, would be eligible to get the vaccine:

Does she go now? Or does she not, because of her age? And that was very confusing (…) We made a pharmacy appointment and then found out that the age-eligibility, though it wasn’t worded right, seemed to override [her disability], so we didn’t get the shot at the pharmacy (Sydney’s caregiver)

These information gaps were a source of frustration for many participants, who were left confused about their eligibility for a COVID-19 vaccine or not knowing how to access one. One participant living with a chronic illness and chronic pain explained that the priority groups were not adequately communicated.

And these [priority group] changes are so crazy that you can’t keep up with them. And I feel sorry for people who maybe aren’t technologically savvy or don’t have access to internet all the time to keep checking these changes, especially rural Manitoba, internet is a huge issue. So, a lot of people are left in the dark, and especially people with disabilities. If they don’t have the worker there to give them the updates or tell them, you’re literally navigating a system with no light to see. And it’s challenging (Jennifer).

Some participants criticized the provincial vaccine rollout and the priority groups. The province prioritized people by age and underlying conditions, so that the elderly and those with higher probability of serious illness got the vaccine first. However, participants noted that requiring both—advanced age and a condition—meant that younger people with disabilities were left vulnerable. One participant argued that “younger people who are vulnerable or even compromised should have been prioritized early on” (Kathryn). Another participant described a situation in which a person with a disability “were too old to be in priority list one [but] too young to [go to the mass vaccination] clinics. So, they kind of fell in between” (Chelsey).

#### Physical access to vaccination sites

Participants recalled feeling anxious about going to their appointment, due to the vaccine itself and uncertainty about accessibility. One participant with both physical and visual impairments explained she immediately thought about all the barriers she would encounter:

I’m going to have to [go] around some physical barriers with my scooter because they section off the sidewalk in front of some of the sites and I have to figure out how to get to the front door. And getting to where I need to actually get the vaccine is usually a wide-open space. I also haven’t had anyone offer large print consent forms or whatever, so I’ve just had people help me if I needed it. But it doesn’t feel for me like it’s welcoming or easy to maneuver (Paula).

Another participant with a chronic disease and mobility issues who requires ongoing oxygen therapy explained feeling anxious about going to his vaccine appointment the following day, as the vaccine site wait-time was likely to outlast his oxygen supply. This particular anxiety arose because the province suddenly opened the vaccination site to same-day walk-ins, and he worried about how pre-booked appointments would be managed alongside walk-ins:

I’m not going to [my COVID vaccine appointment] (…) I use Handi Transit and because I only have a couple of hours of oxygen I can’t be waiting in line, you know. And I can’t be around crowds of people. And so, I think that there was a big mistake that they made (Len).

Several participants indicated they did not have access to a vehicle, and that they were hesitant to expose themselves to greater risk by taking public transit. Many of these participants had to rely on friends or relatives to take them to vaccine appointments. One participant recalled that “a friend of mine, she’s low vision, she had an appointment, somebody was going with her, they couldn’t make it, so she had to cancel her appointment and reschedule it” (David). Another participant described long wait-times, queues, and distances at the vaccination sites as further barriers. A participant who cannot walk long distances explained that:

[A]ccessibility to these sites is not the best (…) Having to walk that line to get somewhere, they should have accounted for that type of mobility issue, (…) getting into even the handicapped area of that [mass vaccination centre] was a real challenge (Darryl).

#### Vaccination experience

Besides having to overcome physical barriers to access a COVID-19 vaccine, participants described several situations during their vaccine appointments that made the experience less inclusive. Participants mentioned that, due to having compromised immune systems, they could not be in crowded spaces such as the mass vaccination clinics.

The d/Deaf participants also mentioned that ASL interpreters were not always available at vaccination sites, as explained by Terri:

I noticed with the deaf community […] we need an interpreter, especially for the vaccines and programs. Some deaf people, they’ve been sharing their experience about when they went and got the vaccine. And they’re saying well, I don’t really need an interpreter, it was great. But then sometimes the interpreters are not always available, so that’s been tough.

Many participants expressed frustration about having to navigate the vaccination sites with little help, a lack of privacy, and having trouble completing the consent forms for various reasons. For example, a participant explained that “I had a hard time filling out the form. I did mine at a pharmacy and there wasn’t any privacy because you’re sitting beside another person that’s doing the form too” (Valerie). This frustration was echoed by another participant who found the consent form hard to fill out:

I have a very hard time holding a pen or using a pen. I asked about having someone else check boxes for me and they said they couldn’t do that, that I needed to do it myself. I got through it but it was very difficult (…) It’s a small thing, but it would have made the day a little bit easier, like between the distance that I had to walk and the time I had to stand and the pen issue, none of it was especially easy (Josy).

Citing difficulties ranging from a lack of information, to physical barriers, transportation challenges, and a lack of accommodation for their specific disabilities, participants described vaccination experiences that were far from accessible and generated unnecessary difficulties and anxiety.

### Recommendations to increase equitable access to the vaccine

Participants noted that many of the obstacles identified above could have been prevented through standard accessibility practices, such as ensuring vaccination sites are wheelchair accessible, chairs are available, and online and print documents are offered in a variety of formats. However, a number of accessibility considerations would have required the involvement of the PLWD community before the vaccination program launch to ensure the needs of the community were met.

#### Vaccine information and booking an appointment

Many participants indicated that finding information on the COVID-19 vaccine was difficult. They suggested making information on accessing vaccine programs clear and straightforward for PLWD. Additionally, participants with low vision recommended that websites containing important information be plain-text and concise so those with screen-readers can navigate them with ease.

Participants were met with various issues booking a vaccination appointment and suggested several improvements such as allowing third-person booking, ensuring online consent forms are form-fillable for those with screen-readers, and allowing the option of booking online or by phone. As participants explained, solely relying on telephone booking creates barriers for those who are d/Deaf while solely depending on online booking systems creates barriers for those using screen-readers or those with unreliable internet access.

Consistently, participants noted the need for an option to request accommodations when booking a vaccine. One participant suggested, and others echoed the sentiment, that these options could be easily incorporated into booking. For example,

I think setting up a separate [phone line], or a separate booking site online, for people to do that, they can tick off the boxes for where they need those supports. So that way, when they go for that appointment, those supports are there for them (Jennifer).

Many emphasized the importance of involving trusted community leaders to distribute information about vaccines, initiate conversations with vaccine-hesitant community members, and help more people get vaccinated. Making information more readily available and providing specific resources such as a phone line or booth at the vaccine site for the public to ask questions, get further information, and ultimately take their time with such an important process were much needed but absent resources.

I feel like a dedicated phone number would be smart (…) there should be an easier way to find answers than having to dig around. And I feel like a pharmacy should be able to answer more questions or give out pamphlets as well. (…) That would be pretty helpful (Chelsea).

Participants expressed the need to have more community vaccination sites (with the involvement of community leaders and organizations) and mobile vaccination teams to go to people’s homes or workplaces.

“[I]t would have been nice if more pharmacies and clinics and even like maybe some Community Centres maybe offered you know pop-up clinics. Or, if they had gotten the process of the mobile vans, or the home visits started sooner. (…) But if they had expanded the options a bit more, it could have been helpful for people like me who have limited transportation resources and limited people I can ask to go with me” (Ana).

Mobile vaccination teams would reduce barriers not only for PLWD but also for low-income people and others facing barriers to getting vaccinated.

#### Improving accessibility to vaccination sites

As previously noted, extensive walking, crowds, and long wait times at vaccine sites proved difficult for many PLWD and serve as further rationale for bringing vaccines to people’s homes and adding more community vaccination sites. Participants from smaller urban centres and rural areas noted they would benefit from vaccination clinics in these areas or even having mobile vaccination clinics, as getting the COVID-19 vaccine often requires traveling long distances to urban areas, which is particularly difficult for older people and those reliant on others for transportation.

Participants emphasized the need for vaccination sites to be fully accessible in terms of built environment considerations that facilitate accessibility for people using walkers or wheelchairs, and/or those with vision impairments. In addition, they highlighted the need for a variety of formats (e.g., large-print, braille) of consent forms and take-home information on the vaccine and possible side effects. Support may also be required in filling out the consent forms, whether for people with intellectual disabilities, those with musculoskeletal conditions who may not be able to hold a pen, or those who otherwise have difficulties filling in forms.

#### Improving vaccination experience

Referring to their vaccination experience, participants indicated a general lack of understanding towards PLWD by vaccination centre staff. The Manitoba Accessibility Office provided vaccination sites with resources on accessible customer service, yet participant experiences indicate a lack of implementation of such guidelines. It is critical that vaccination site staff have (and make use of) appropriate resources to support PLWD. Participants recounted interactions where vaccination site staff questioned the presence of a support person, communicated to support workers instead of the participant themselves, and grew impatient when participants required extra time to complete a task or requested further information about the vaccine. Many participants emphasized that hiring PLWD to help others navigate vaccination sites would improve the overall experience.

Valerie: They should hire some of us with disabilities to work there so we can help navigate other people with disabilities.

David: That does make sense because there would be a deeper level of understanding what that individual is going through, what their needs may be. So, yeah, that is important, I would go for that.

Participants added that staff at vaccination sites should also be prepared to help people who might need more direct support. A participant with vision impairments explained “I went to the [mass vaccination centre] to get my vaccination and being totally blind there’s no way I could navigate that building on my own.” The participant recommended having staff trained to assist visually impaired people at vaccination sites. Drawing on his own experience training retail workers to assist visually impaired customers, he indicated that there are “proper and safe guiding techniques that they [can] apply in general [and] even more now that we’re in social distancing times.” Such training helps reduce anxiety for visually impaired people accessing public spaces, and allows staff to feel better “prepared and not as nervous as they would be without that education” (David). Further, sign language interpreters should be available for d/Deaf individuals from the moment they arrive at the vaccination site. Staff should be prepared to use alternative communication strategies, such as writing back and forth, if needed.

Finally, there is a need to offer more privacy during the vaccination process. A participant explained that she took her child with disabilities to get vaccinated, and the staff were loud and condescending when her child took extra time to answer routine questions. This compelled the participant to disclose her child’s status as a person living with intellectual disabilities in an effort to get the staff to respond more patiently:

[M]y daughter paused on her birthday, you know, she was thinking in her head, and the lady was so rude. She said, “You don’t know your birthday?” And I kind of got a little bit taken back and (…) I said, “I’ll have you know, you’re speaking with person who has disabilities” (…) And it really put me off because I got thinking, how are other people being talked to who have disabilities, whether they be intellectual or physical? (Jennifer).

## Discussion

The COVID-19 vaccination program in Manitoba has not been fully accessible for people living with disabilities. In our focus groups, most participants expressed frustration about their vaccination experience and identified a series of barriers, including (1) lack of access to information about the vaccine and vaccination program; (2) challenges with the phone and online booking systems to make a vaccination appointment; (3) difficult physical access to vaccination sites; (4) difficulty navigating the vaccination sites with little assistance; and (5) lack of privacy and accommodations. While some of these barriers were addressed as the vaccination program progressed, many of these problems could have been avoided had PLWD been engaged in program planning from the outset.

Adopting public health strategies with a focus on disability justice (Berne et al., 2018; Piepzna-Samarasinha, 2018) would improve access to, and experiences of, vaccination for PLWD and could potentially help other populations whose social locations often intersect with disability (e.g., BIPOC, low income, precariously employed, seniors). While participants did not explicitly comment on gender-, race-, or class-related barriers, the importance of an intersectional approach is reflected through varied concerns around the intersections of age, disability, and geographic location (Crenshaw, 1990; Berne et al., 2018). The “single-issue” approach to vaccine programs (e.g., age-based eligibility, online-only booking, centralized vaccine sites) failed to meet the needs of the most marginalized and therefore most vulnerablized (Berne et al., 2018; Tremain, 2020). Similarly, participants living with multiple disabilities experienced layered and sometimes conflicting access needs that would not be captured through a “checklist” approach to accommodations. Considering the complexities and nuances of participants’ lived experiences accessing vaccines, it is clear that the disability justice tenet of “leadership of the most impacted” is greatly needed (Berne et al., 2018). Meaningful engagement with a diverse population of PLWD in immunization program planning would help ensure that PLWD, who already face significant challenges due to COVID-19 (Lebrasseur et al., 2021), are offered the same protections from disease as the rest of the population.

As a single recommendation for vaccination programs, participants supported hiring and engaging PLWD in the entire vaccine navigation process. This recommendation echoes the disability rights movement mantra: “nothing about us without us” (Charlton, 1998). PLWD are better equipped to assist other PLWD based on their shared experiences—a concept disability justice leaders refer to as “crip emotional intelligence”^4^ (Piepzna-Samarasinha, 2018)—and can provide guidance about accommodations more generally to improve immunization program access.

Dedicating resources to providing accommodations for PLWD would allow vaccination sites to be more accessible and create better conditions for everyone. Through the involvement of PLWD who are experienced in implementing accessibility practices, vaccination programs can employ an “active offer” of accommodations (Manitoba, 2013). An active offer communicates an understanding of accessibility and can serve as an opportunity for dialogue on how to best meet an individual’s needs.

Training staff at vaccination sites to accommodate PLWD is a crucial step to ensure vaccine equity, particularly by showing patience and sensibility, as well as ensuring privacy. However, training must not only equip staff to provide specific accommodations but also generally increase their knowledge about PLWD. PLWD could be involved in the training process to facilitate a nuanced understanding of the challenges PLWD face in accessing services—like that of the COVID-19 vaccination program. Training would aim to prevent staff from treating PLWD as “people [they] must begrudgingly provide services for” (Piepzna-Samarasinha, 2018, p. 76) and instead, respect PLWD as valuable members of the community. Additionally, mobile vaccination teams were suggested to reduce barriers for PLWD. This initiative could improve vaccine access for low-income communities and others facing barriers to getting vaccinated, reflecting the importance of an intersectional approach to vaccination programs. Further involvement with localized community health centres would also help address these barriers by decentralizing the vaccination process.

### Limitations

Our study has three main limitations. First, we focused on the experiences of PLWD in Manitoba and our results are not generalizable to all PLWD. However, a significant strength of the study is the cross-disability focus which helps counter this limitation. The second limitation of our study is the limited sample size, which does not represent all types of disabilities and some categories of disability (e.g., intellectual disabilities and physical impairments) had only one or two participants. Nonetheless, where participants disclosed more than one disability in their narratives, these were included in the results. However, identifying a primary disability experience was a function of our survey instrument. Future studies could expand participant recruitment to increase representation across disabilities and in other jurisdictions, to further our understanding of the barriers faced by PLWD. Additionally, future studies could also examine how the intersections of gender, class, race, and Indigenousness affect the experiences of PLWD. While we had one participant self-disclose as Métis, no other participant did so and participants were not specifically recruited for Indigeneity. Within our larger study, however, we will be exploring the experiences of Red River Métis in Manitoba through a partnership agreement with the Manitoba Métis Federation. Finally, we document experiences of PLWD during a time when the vaccine program was undergoing continual changes. Some barriers identified early on were corrected by public health authorities. Despite these limitations, the experiences identified in this study are likely comparable to the experiences of PLWD in other Canadian jurisdictions where vaccine programs underwent similar challenges; however, this would require further study.

## Conclusion

We suggest that a public health response oriented to disability justice would remedy many of the inequities outlined by our participants. Of utmost importance is a commitment to listening to and collaborating with PLWD in the vaccination planning process to prevent undue hardship for PLWD accessing vaccinations. These recommendations could easily be applied to current and future immunization programs, including those beyond Canada, to increase accessibility for all.

## Contributions to knowledge

What does this study add to existing knowledge?

- We identify barriers to the COVID-19 vaccine for people living with disabilities (PLWD), which were not accounted for by provincial and public health authorities.
- PLWD referred to three areas where they encountered barriers: (1) vaccine information and appointment booking, (2) physical access to vaccination sites, and (3) overall experience.
- Some of the barriers identified (e.g., lack of wheelchair accessibility, lack of chairs on-site) were easily predictable and avoidable. Others could have been prevented by engaging PLWD in the planning stage of the vaccination program (e.g., allowing third-party appointment booking, requesting accommodations when booking appointments, and training staff to engage PLWD with respect and accommodate them).

What are the key implications for public health interventions, practice, or policy?

- The most critical recommendation participants made is to include and engage PLWD in the vaccination planning process and seek out PLWD to work in vaccination clinics.
- We suggest that a public health response oriented to disability justice would remedy many of the inequities faced by PLWD. Of utmost importance is a commitment to listening to and collaborating with PLWD in the vaccination planning process to prevent undue hardship for PLWD accessing vaccinations.
- Other recommendations include making all information and documents accessible in various ways, increasing mobile vaccination teams, ensuring individuals can request accommodations for their vaccine appointments, and training staff to handle accommodations for PLWD adequately.
- Our findings and recommendations can inform vaccination processes around the world, particularly those involving children living with disabilities, to avoid the anxiety and trauma described by our focus group participants.

## Data Availability

Focus group guides are available upon request.

## Footnotes

1 We consulted with The Independent Living Resource Centre (Winnipeg), Manitoba Accessibility Office, Manitoba League of Persons with Disabilities, and the Council of Canadians with Disabilities (Manitoba chapter).

2 Including gender, age, race, ethnicity, marital status, education level, income, and type of disability. There were five disabilities participants could choose from: mental illness/mental health condition; chronic illness/pain; sensory impairment including hard of hearing or d/Deaf; intellectual disability; physical impairment.

3 First thoughts about COVID-19; Testing and contact tracing; Vaccines (general); COVID-19 vaccines; Priority groups; Acceptability of the COVID-19 vaccine; (Dis)Trust in Information Sources.

4 “Crip” is a term associated with radical disability culture and politics. Although its use is not without contestation, it refers to a practice of reclaiming slurs aimed at people with disabilities and re-imagining cultural narratives of disability. Piepzna-Samarasinha’s (2018) coupling of ‘crip’ with ‘emotional intelligence’ highlights the “particular skills, talents, sciences, and cultures” (p.69) that disabled folks develop through their experience of disability. Thus, the notion of crip emotional intelligence counters the “deficiency model by which most people view disability” (p.69).

## Code availability

Coding guides are available upon request.
